# Factors associated with complications in ST-elevation myocardial infarction: a single-center experience

**DOI:** 10.1186/s12872-023-03498-z

**Published:** 2023-09-19

**Authors:** Jean-Michel Mavungu Mbuku, Aldophe Mukombola Kasongo, Pascale Goube, Laetitia Miltoni, Aliocha Nkodila Natuhoyila, Jean-Réné M’Buyamba-Kabangu, Benjamin Longo-Mbenza, Bernard Kianu Phanzu

**Affiliations:** 1 Unit of cardiology, University of Kinshasa, 58, Avenue Biangala, Righini, Commune Lemba, Kinshasa, Democratic Republic of Congo; 2 Cardiology Intensive Care Unit, Hôpital Sud Francilien, Paris, France; 3 Department of Biostatistics, Public Health School of Kinshasa, Kinshasa, Democratic Republic of Congo

**Keywords:** STEMI, Frequency, Determinants, Complications, Sud Francilien Hospital Center

## Abstract

**Background:**

ST-elevation myocardial infarction (STEMI) is a major public health problem. This study aimed to determine the prevalence and identify the determinants of STEMI-related complications in the Cardiology Intensive Care Unit of the Sud Francilien Hospital Center (SFHC).

**Methods:**

We retrospectively analyzed the data of 315 patients with STEMI aged ≥ 18 years. Logistic regression was used to identify factors independently associated with the occurrence of complications.

**Results:**

Overall, 315 patients aged 61.7 ± 13.4 years, of whom 261 were men, had STEMI during the study period. The hospital frequency of STEMI was 12.7%. Arrhythmias and acute heart failure were the main complications. Age ≥ 75 years (adjusted odds ratio [aOR], 5.18; 95% confidence interval [CI], 3.92–8.75), hypertension (aOR, 3.38; 95% CI, 1.68–5.82), and cigarette smoking (aOR, 3.52; 95% CI, 1.69–7.33) were independent determinants of acute heart failure. Meanwhile, diabetes mellitus (aOR, 1.74; 95% CI, 1.09–3.37), history of atrial fibrillation (aOR, 2.79; 95% CI, 1.66–4.76), history of stroke or transient ischemic attack (aOR, 1.99; 95% CI, 1.31–2.89), and low high-density lipoprotein-cholesterol (HDL-C) levels (aOR, 3.70; 95% CI, 1.08–6.64) were independent determinants of arrhythmias.

**Conclusion:**

STEMI is a frequent condition at SFHC and is often complicated by acute heart failure and arrhythmias. Patients aged ≥ 75 years, those with hypertension or diabetes mellitus, smokers, those with a history of atrial fibrillation or stroke, and those with low HDL-C levels require careful monitoring for the early diagnosis and management of these complications.

## Background

The prevalence of cardiovascular diseases (CVDs) has almost doubled in the previous three decades, from 271 million in 1990 to 523 million in 2019. Mortality from these diseases also increased steadily during this period, from 12.1 million in 1990 to 18.6 million in 2019 [1],which is one-third of the total global mortality [1–3].

Ischemic heart disease (IHD) is undoubtedly the most common form of CVD [4]. Despite tremendous achievements in its management, IHD remains one of the leading causes of premature death, disability, and human suffering worldwide [4, 5]. The global prevalence of IHD is rising, with the 2017 prevalence rate of 1,655 per 100,000 individuals expected to exceed 1,845 by 2030 [5]. Furthermore, IHD is recognized as a real threat to sustainable development in the 21st century [6].

Acute coronary syndrome (ACS) is the most severe and life-threatening manifestation of IHD, with an annual incidence of approximately 7 million worldwide [7].

Since the European Society of Cardiology (ESC)/American College of Cardiology Foundation/American Heart Association/World Heart Federation expert consensus of 2012 [8], ACS has been distinguished into two main types: ST-elevation myocardial infarction (STEMI) and non-ST-elevation myocardial infarction (non-STEMI). The two categories have differences in terms of clinical manifestations, pathology, pathogenesis, treatment, and prognosis [9–13]. The study by Vernon et al., demonstrating a substantial increase in the number of patients with STEMI without standard modifiable cardiovascular risk factors (CVRFs) [14], has shown that there remain gray areas in our understanding of the pathogenic mechanisms of STEMI. Besides, a recent French population-based registry analyses from the Monitoring of Trends and Determinants in Cardiovascular Disease (MONICA) project has shown that the vital prognosis is worse in the short term in patients with STEMI than in those with non-STEMI [15].

The emergence of new therapies, particularly the advent of thrombolytics, the rise of coronary units, the development of pre- and per-procedural pharmacological support, and the organization of pre- and intra-hospital care have significantly reduced hospital mortality from STEMI [16–18].

Despite this recent decrease in mortality, STEMI remains a diagnostic and therapeutic emergency involving short-, medium-, and long-term vital prognosis and requires early, rapid, and appropriate management [19].

The complexity of acute STEMI management lies in the many unexpected complications that can enamel its evolution, particularly heart failure [20], rhythmic disorders [21, 22], and mechanical complications [23]. These complications decline over time, without completely disappearing, with the advent of the aforementioned new management strategies, and are always accompanied by an exceptionally high mortality rate and are one of the main causes of death in the early phase following MI [24].

Nowadays, due to the revolution and modernization of ACS management, only a few studies have addressed these complications and resulted in divergent results, both for the frequency and for the factors associated with the occurrence of these complications; some of the most cited factors for the occurrence of these complications were as follows: race/ethnicity [25, 26] and sex disparities [26]; the existence of chronic kidney disease; diabetes mellitus; high total cholesterol, low-density lipoprotein (LDL) cholesterol, and high-sensitivity C-reactive protein levels [27]; prior heart failure; anemia; multivessel disease; and anterior location [28].

Not only the different methodological approaches used in these different studies but also the large regional variation in clinical profiles demonstrated in patients with STEMI [29] could explain these discrepancies.

Of note, most studies have addressed both STEMI and non-STEMI simultaneously. In the scientific literature, information on both the frequency and determinants of acute complications specific to STEMI in Essonne, France, is lacking. Thus, this study aimed to determine the in-hospital prevalence and factors associated with the occurrence of acute complications of STEMI. This information could be considered in developing decision-making algorithms for managing STEMI and could contribute to reducing mortality.

## Methods

### Study design and setting

This retrospective single-center analysis of the records of patients with STEMI admitted to the Cardiology Intensive Care Unit (CICU) of the Sud Francilien Hospital Center (SFHC), in the French Republic, was conducted from January 1, 2020 to December 31, 2021. The SFHC is located in Essonne, in the cities of Corbeil-Essonnes and Yerres. It provides hospital coverage for approximately 600,000 inhabitants (all of Essonne, south of Seine-et-Marne, and southeast of Val-de-Marne, France).

### Sample size calculation

A sample size of 322 was estimated taking the frequency of STEMI as 40% of French MONICA registries [30] (confidence level = 95% and margin of error = 5%).

### Patient selection

This study included all men and women aged at least 18 years who had been hospitalized for STEMI between January 1, 2020 and December 31, 2021.

Patients admitted for STEMI but who arrived ≥ 24 h after the onset of symptoms and those whose medical records lacked variables of interest were excluded from the study.

### Study procedures

All medical records of the patients admitted for STEMI were retrieved and carefully reviewed by two researchers (JMMM and AK) to obtain relevant data on the parameters of interest, which included sociodemographic data (i.e., age, sex, and occupation); CVRFs (i.e., hypertension, diabetes mellitus, dyslipidemia, cigarette smoking, obesity, and coronary heredity); history (i.e., IHD, obliterating arterial disease of the lower limbs, carotid angioplasty or coronary artery bypass surgery, heart rhythm disorder, stroke/TIA, heart failure, chronic renal failure, neoplasia, pulmonary embolism, and coronavirus disease 2019 [COVID-19]); clinical data (i.e., blood pressure, heart rate, and oxygen saturation); electrocardiographic aspects (i.e., ST segment elevation, presumed recent left bundle brunch, rhythm, and conduction disorders); echocardiographic characteristics (i.e., left ventricular ejection fraction using Simpson’s biplane method and kinetic disorders); biological data (i.e., serum creatinine, renal creatinine clearance according to the Modification of Diet in Renal Disease equation, glycated hemoglobin (HbA1c), total cholesterol, LDL-cholesterol, high-density lipoprotein [HDL]-cholesterol, and triglycerides); coronary angiographic lesions (i.e., type and number of occluded coronaries [i.e., monotroncular, bitroncular, and tritroncular]); the number of stents implanted; rhythmic and conductive post-coronarography disorders (i.e., atrial, junctional, and ventricular arrhythmias); and hemodynamic (i.e., acute heart failure with or without acute pulmonary edema or cardiogenic shock), mechanical and embolic (i.e., ischemic stroke) complications.

All patients underwent primary percutaneous coronary intervention and were continuously evaluated and monitored for temperature, systolic and diastolic blood pressure, SpO2, heart rate frequency, and 12-lead electrocardiogram ECG within 24 h of admission to the CICU. These parameters were remotely monitored using a multi-parameter portable vital signs monitor (BeneVision N22/N19, Mindray, Mahwak, USA). Moreover, a follow-up examination was performed, which included symptoms (such as dyspnea, chest pain, and palpitations) and biological parameters (such as complete blood count, creatinine, ionogram, glycaemia, troponin, creatinine phosphokinase, C-reactive protein, prothrombin time, and activated partial thromboplastin time). All complications from the initial contact with emergency medical services up 24 h after the admission to the CICU were considered in this study. All patients were treated according to the ESC guidelines for managing acute MI in patients presenting with ST elevation [31].

### Operational definitions

The following definitions were used in this study:

According to the 2017 recommendations of the ESC [31], STEMI was defined as chest pain associated with the following: A new ST elevation at the J point in at least two contiguous or adjacent leads: in V2–V3 ≥ 0.2 mV (2 mm) in men after 40 years (≥ 0.25 mV before 40 years)) and ≥ 0.15 mV (1.5 mm) in females or ≥ 0.1 mV (1 mm) in other leads; a left bundle branch block in the presence of the Smith criteria [32, 33]; or new ST depression from V1 to V3 (V4) on a 12-lead tracing, associated with ST elevation ≥ 0.5 mm in at least two leads from V7 to V9.

Dyslipidemia was defined as an LDL-cholesterol level ≥ 1.6 g/L and/or an HDL-cholesterol level ≤ 0.40 g/L in men and ≤ 0.50 g/L in women and/or a total cholesterol level ≥ 2 g/L and/or a triglyceride level ≥ 1.5 g/L [34]. LDL-cholesterol was calculated using the Friedewald method as LDL-cholesterol (g/L) = CT (g/L) − HDL-cholesterol (g/L) − triglyceride (g/L)/5 (if the triglyceride level was ≤ 3.4 g/L) [34, 35]. It was directly dosed using the dextran sulfate filtration technique if the triglyceride level was > 3.4 g/L [34, 36].

Left ventricular ejection fraction (LVEF) values were categorized into three groups according to the 2021 ESC recommendations [37], as follows: “reduced” when the LVEF was ≤ 40%; “mildly reduced” when the LVEF was between 40% and 49%; and “preserved” when the LVEF was ≥ 50%.

### Statistical analyses

The qualitative data are represented as absolute (n) and relative frequencies (%), and the quantitative data are presented in the form of means ± standard deviations (if the distribution is normal) or medians with their quartile interquartile ranges (if the distribution is asymmetric). The distribution of each variable was assessed using the Kolmogorov–Smirnov test. Simple logistic regression was used to determine which factors were predictive of complications. The following variables were entered into the univariate analysis: age group (< 55, 55–75, and > 75 years), CVRFs (i.e., hypertension, diabetes mellitus, dyslipidemia, obesity, cigarette smoking, and coronary heredity), medical history (i.e., stented IHD, coronary artery bypass surgery, previous atrial fibrillation, previous stroke, heart failure, chronic kidney disease, peripheral artery occlusive disease, neoplasia, pulmonary embolism, and COVID-19), the number of occluded vessels (i.e., monovessel, bivessel, and trivessel), and LVEF (i.e., reduced, moderately reduced, and preserved). The odds ratios (ORs) and their 95% confidence intervals (95% CIs) were finally calculated to assess the degree of association between the variables and occurrence of complications. When the associations were observed between complications and these independent variables, the effects of potential confounders were examined by adjustment in a conditional logistic regression (multivariate analysis). The significance threshold retained was *p* < 0.05. All data were analyzed using Statistical Package for the Social Sciences (version 24; IBM Corp., Armonk, NY).

## Results

### Characteristics of the study population

The study population consisted of 315 patients, with a mean age of 61.7 ± 13.4 years; of the 315 patients, 261 were men and 54 were women, with a sex ratio of 4.8 (in favor of men).

### Hospital frequency of STEMI

Overall, 2,479 patients were hospitalized in the CICU-SFHC during the study period. Among them, 315 presented with STEMI, with a frequency of 12.7%.

### Sociodemographic characteristics of the study population according to sex

Table 1 shows the sociodemographic characteristics of the population. The mean age of patients was 61.7 ± 13.4 years, and 47.6% were aged 55–75 years. The same table shows that retirees were the most represented professional category.

**Table 1:** Sociodemographic characteristics of the study population

Variable	Frequency	Percentage
Age group		
< 55	104	33.0%
55–75	150	47.6%
> 75	61	19.4%
Sex		
Male	261	82.9%
Female	54	17.1%
Professional categories		
Management and intellectual professions	26	8.3%
Employees	18	5.7%
Workers	66	21.0%
Unemployed	27	8.6%
Intermediate categories	33	10.5%
Retirees	154	46.0%

### CVRFs of the study population

As illustrated in Table 2, the main CVRFs were cigarette smoking (58.4%), hypertension (42.9%), dyslipidemia (25.4%), and obesity (19.4%).

**Table 2:** Distribution of cardiovascular risk factors

Variable	Frequency	Percentage
Hypertension	13	42.9%
Diabetes	62	19.7%
Dyslipidemia	80	25.4%
Obesity	61	19.4%
Cigarette smoking	184	58.4%
Coronary heredity	32	10.1%

### Medical history

The patients’ medical history is shown in Table 3. It was dominated by stented IHD (15.2%), followed by neoplasia (7%), atrial fibrillation (3. 5%), and chronic kidney disease (2.5%).

**Table 3:** Patients’ medical history

Variable	Frequency	Percentage
Stented IHD	48	15.2%
Coronary artery bypass surgery	3	1.0%
AF	11	3.5%
Stroke/TIA	7	2.2%
Heart failure	2	0.6%
CKD	8	2.5%
PAOD	6	1.9%
Neoplasia	22	7.0%
Pulmonary embolism	2	0.6%
COVID-19	1	0.3%

AF, atrial fibrillation; COVID-19, coronavirus disease 2019; CKD, chronic kidney disease; IHD, ischemic heart disease; PAOD peripheral arterial occlusive disease; TIA, transient ischemic attack

### Coronagraphic characteristics of the patients

The coronary angiographic characteristics of the patients are shown in Table 4. Monovessel lesions were the most common (59%), followed by bivessel lesions (27.3%) and trivessel lesions (13.7%). The left anterior descending artery (LADA) was the most often affected artery (49.46%) in patients with monovessel lesions. Regarding bivessel lesions, the LADA and right coronary artery (RCA) were the most often affected blood vessels (44.18%). As for trivessel lesions, the LADA, left coronary artery, and RCA was the most affected blood vessels (58.13%).

**Table 4:** Coronagraphic characteristics of the patients

Variables	Frequency	Percentage
Monovessel lesion	186	59.0%
LADA	92	49.46%
RI	2	1.08%
RC	54	29.03%
LCA	22	11.83%
D	6	3.22%
PDA	1	0.54%
OM	6	3.22%
PLA	1	0.54%
CT	2	1.08%
**Bivessel lesions**	**86**	**27.3%**
RCA and RI	1	1.16%
RCA and D	4	4.65%
RCA and OM	1	1.16%
LCA and RCA	5	5.81%
LCA and D	2	2.32%
D and OM	1	1.16%
**LADA and RCA**	**38**	**44.18%**
LADA and LCA	21	24.41%
LADA and OM	4	4.65%
LADA and RI	3	3.49%
OM and RCA	3	3.49%
PLA and D	1	1.16%
LADA and RCA	1	1.16%
RCA and D	1	1.16%
**Trivessel lesions**	**43**	**13.7%**
LCA, OM, and RI	1	2.33%
LCA, OM, and RCA	1	2.33%
LCA, RCA, and D	2	4.65%
LADA, OM, and LCA	1	2.33%
LADA, RI, and LCA	1	2.33%
LADA, OM, and RCA	3	6.98%
LADA, OM, and PDA	1	2.33%
LADA, OM, and RCA	4	9.30%
LADA, OM, and D	1	2.32%
LADA, RI, and RCA	2	4.65%
**LADA, LCA, and RCA**	**25**	**58.13%**
OM, RC, and D	1	2.33%

LADA, left anterior descending artery ; RI, ramus intermedius ; RC, right coronary ; LCA, left coronary artery ; D, diagonal; PDA, posterior descending artery ; OM, obtuse marginal ; PLA, posterolateral artery ; RCA, right coronary artery

### Echocardiographic characteristics

The distribution of the patients’ echocardiographic findings is reported in Table 5. The mean LVEF was 50.61 ± 10.9%. Furthermore, 62.86% of the patients had an LVEF ≥ 50%, 24.13% had an LVEF between 40% and 49%, and 13.02% had an LVEF ≤ 40%. Overall, segmental kinetics disorders predominated in the anterior parts (41.9%) followed by the inferior parts (38.71%).

**Table 5:** Echocardiographic characteristics of the patients

Variables	Frequency	Percentage
**LVEF (%)**	50.61 ± 10.9	
≥ 50%	198	62.86%
40–49%	76	24.13%
< 40%	41	13.02%
**Kinetic abnormalities**		
Anterior akinesia	2	0.63%
Antero-septal akinesia	3	0.95%
Apical akinesia	40	12.69%
inferior akinesia	18	5.71%
Inferior lateral akinesia	3	0.95%
Septo-apical dyskinesia	1	0.32%
Apical hypokinesia	28	8.89%
Anterior hypokinesia	16	5.08%
Antero-lateral hypokinesia	9	2.86%
Antero-septal hypokinesia	33	10.48%
Inferior hypokinesia	77	24.44%
Inferior-lateral hypokinesia	22	6.98%
Inferior septal hypokinesia	3	0.95%
Lateral hypokinesia	2	0.63%
Global hypokinesia	3	0.95%
No kinetic disorder	55	17.46%

LVEF, left ventricular ejection fraction

### Clinical and biological characteristics of the patients

The clinical and biological characteristics of the patients are shown in Table 6. On admission, the average values for the biological parameters, systolic and diastolic blood pressure, heart rate, and oxygen saturation were all within the normal range.

**Table 6:** Clinical and biological characteristics of the patients

Variables	M ± SD
SBP (mmHg)	121.9 ± 22.4
DBP (mmHg)	74.4 ± 13.8
Heart rate (bpm)	77.8 ± 14.5
SpO2 (%)	97.7 ± 2.1
HbA1c (%)	6.1 ± 1.36
Creatinine (µmol/L)	86.8 ± 29.8
GFR (mL/min/1.73 m^2^)	91.28 ± 27.56
Na+ (mmol/l)	138,1 ± 3,7
K+ (mmol/l)	3,9 ± 0,4
HDL-c (mg/dL)	45.2 ± 19.4
LDL-cholesterol (mg/dL)	120.0 ± 41.6
Total cholesterol (mg/dL)	78 ± 47
Triglycerides (mg/dL)	141.5 ± 8.6

DBP, diastolic blood pressure; GFR, glomerular filtration rate; HbA1c, glycated hemoglobin; HDL-C, high-density lipoprotein cholesterol; K+, potassium; LDL-C, low-density lipoprotein cholesterol; Na+, sodium; SBP, systolic blood pressure; SpO2, oxygen saturation; WBC, white blood cells

### Kinetics of cardiac enzymes

The cardiac enzyme kinetics of all patients studied are present in Table 7. Notably, troponin levels at admission increased seven-fold before subsequently decreasing, whereas creatinine phosphokinase levels tripled before decreasing.

**Table 7:** Cardiac enzyme kinetics

Cardiac enzyme	At admission *median (p25-p75)*	At the peak *median (p25-p75)*	At discharge *median (p25-p75)*
Troponine (pg/ml)	350 (77.5–1202)	2550 (960–4500)	705 (286–1491)
CPK (U/l)	245 (121-507.3)	771.5 (485–1599)	160 (120–229)

CPK, creatinine phosphokinase

### Distribution of complications

The patient complications are listed in Table 8. Arrhythmias (22.85%) and Killip class ≥ 2 acute heart failure (13.01%) were the most frequent complications.

Among the rhythm disorders, ventricular rhythm disorders accounted for 13.64% and supraventricular rhythm disorders accounted for 9.21%, including atrial fibrillation (8.57%).

**Table 8:** Distribution of the complications

Variable	Frequency	Percentage
Killip class ≥ 2	41	(13.01)
Killip class II	25	(7.94)
Killip class III	10	(3.17)
Killip class IV	6	(1.90)
**Arrhythmia**	72	(22.8)
Atrial flutter	2	(0.64)
Atrial fibrillation	27	(8.57)
uVT	27	(8.57)
SVT	12	(3.80)
VF	3	(0.95)
AIVR	1	(0.32)
**Conduction disorders**		
1st degree AV bloc	4	(1.27)
2nd degree AV bloc Mobitz type 1	1	(0.32)
2nd degree AV bloc Mobitz type 2	2	(0.63)
3rd degree AV bloc	2	(0.63)
**Other complications**		
Tamponade	1	(0.32)
Ischemic stroke	5	(1.59)
Stent thrombosis	1	(0.32)
VSD	1	(0.32)
Coronary dissection	1	(0.32)

AIVR, accelerated idioventricular rhythm; AV bloc, atrioventricular bloc; bloc auriculoventriculaire; VF, ventricular fibrillation; VSD, ventricular septal defect; uVT, unsustained ventricular tachycardia

### Bivariate and multivariate analyses of the factors associated with the occurrence of hemodynamic disorders

Table 9 shows that age ≥ 75 years, hypertension, cigarette smoking, and previous atrial fibrillation were the factors associated with the onset of acute heart failure. After adjustment in the multivariate analysis, only age ≥ 75 years, hypertension, and cigarette smoking emerged as independent determinants of the occurrence of acute heart failure. The risk of developing acute heart failure was increased by five in the presence of an age ≥ 75 years, by three in the presence of hypertension, and by four in the presence of cigarette smoking.

**Table 9:** Bivariate and multivariate analyses of the factors associated with the occurrence of acute heart failure

Variable	Univariate analysis		Multivariate analysis	
	*P*	OR (95% CI)	*P*	aOR (95% CI)
Age				
< 55 years		1		1
55–74 years	0.013	2.88 (1.41–6.95)	0.018	2.50 (1.29–5.74)
≥ 75 years	< 0.001	4.38 (2.78–7.18)	< 0.001	5.18 (3.92–8.75)
Hypertension				
No		1		1
Yes	0.014	2.25 (1.18–4.29)	0.037	3.38 (1.68–5.82)
Cigarette smoking				
No		1		1
Yes	< 0.001	3.41 (1.75–6.66)	0.030	3.52 (1.69–7.33)
Previous AF				
No		1		1
Yes	0.045	1.67 (1.03–3.08)	0.938	1.06 (0.27–4.09)

AF, atrial fibrillation; aOR, adjusted odds ratio; OR, odds ratio

### Bivariate and multivariate analyses of factors associated with arrhythmias

Table 10 shows that diabetes mellitus, stroke history, atrial fibrillation history, chronic kidney disease, and low HDL-C levels were the factors associated with the onset of arrhythmias.

After adjustment in the multivariate analysis, diabetes mellitus, history of atrial fibrillation, history of stroke, and low HDL-C levels persisted as the independent factors for the onset of arrhythmias. Low HDL-C levels multiplied this risk by four, history of AF multiplied this risk by three, and diabetes mellitus and stroke multiplied this risk by two.

**Table 10:** Bivariate and multivariate analyses of the factors associated with arrhythmias

Variable	Univariate analysis		Multivariate analysis	
	*P*	OR (95% CI)	*P*	aOR (95% CI)
Diabetes mellitus				
No		1		1
Yes	**0.024**	2.03 (1.10–3.77)	**0.020**	1.74 (1.09–3.37)
Previous AF				
No		1		1
Yes	**0.023**	3.19 (1.94–10.79)	**0.016**	2.79 (1.66–4.76)
Previous stroke/TIA				
No		1		1
Yes	**0.018**	2.80 (1.61–4.84)	**0.047**	1.99 (1.31–2.81)
CKD				
No		1		1
Yes	**0.022**	3.80 (1.92–5.60)	0.056	1.62 (0.32–2.27)
Low HDL-c				
No		1		1
Yes	**0.017**	3.71 (1.81–6.62)	**0.018**	3.70 (1.08–6.64)

AF, atrial fibrillation; aOR, adjusted odds ratio; CKD, chronic kidney disease; HDL-c, high-density lipoprotein cholesterol; OR, odds ratio; TIA, transient ischemic attack

## Discussion

This study aimed to evaluate the frequency and identify the determinants of complications occurring in patients admitted to the CICU-SFHC for STEMI, in the French Republic, from January 1 to December 31, 2021.

The hospital frequency of STEMI was 12.7%. The main complications were rhythmic (22.85%) and hemodynamic (13.1%). Age ≥ 75 years, hypertension, and cigarette smoking emerged as independent determinants of hemodynamic complications. Meanwhile, diabetes mellitus, history of atrial fibrillation, stroke/TIA, and low HDL-C levels have emerged as independent determinants of arrhythmias.

This is one of the few studies to examine the prevalence of acute complications of STEMI in the era of structured and modernized organization of emergency and cardiac intensive care units.

Before the use of early reperfusion therapy (1960 to 1980 s), the frequency of arrhythmias was elevated following ACS [38–42]. However, a marked decrease in their incidence and an improvement in life expectancy was noted, starting from the late 1980s, with the advent of early reperfusion therapy [43].

Nowadays, arrhythmias and conduction disorders remain among the most common complications of ACS, particularly during STEMI [21]. In this study, 22.8% of the patients had arrhythmias. Other studies have reported an overall incidence of arrhythmias after STEMI as high as 78–83% [44–47]. This difference could be explained by certain characteristics of the patients under study, notably the LVEF, which was reduced in the aforementioned studies. In this study, the average LVEF was 50.61% ± 10.9%. Decrease in the ejection fraction is a well-known significant risk factor for the development of arrhythmias [48]. The duration of follow-up was longer in most aforementioned studies than our study, and the comorbidities and age of the patients enrolled in various studies could also explain the difference in the frequency of arrhythmia, as this frequency increases with the age of patients [49]. According to Gorenek’s report, ventricular arrhythmias and atrial fibrillation were the most common arrhythmias found in this study [50].

The frequency of ventricular arrhythmias found in this study (13.6%) is close to 11% found by Podolecki et al. [51] and the frequency of atrial fibrillation found in this study is similar to that found by other authors [52, 53].

The mechanisms involved in the occurrence of rhythm disorders during ACS include genetic predisposition, which would be an essential condition for early arrhythmogenesis [54], sympathetic nervous system stimulation [55, 56], hypoxia, possible electrolyte imbalances, and damaged myocardium that acts as a substrate for the incoming circuits, due to changes in refractory periods. Furthermore, transmural infarction can interrupt the afferent and efferent branches of the sympathetic nervous system that innervates the myocardium downstream of the infarction zone. This autonomous imbalance promotes arrhythmias and conduction disorders [50, 57].

This study identified diabetes mellitus, history of atrial fibrillation, stroke, and low HDL-C levels as the determinants of arrhythmias.

The finding of diabetes mellitus being a determinant of arrhythmia in STEMI is according to the results from the Thai ACS Registry [58]. A recent study by Karthikeyan has also found diabetes mellitus as a factor associated with STEMI and its complications [27]. Diabetes mellitus is a well-established risk factor for arrhythmias [59–61]. The pathophysiology of diabetes mellitus-related arrhythmias is not fully understood; however, it is related to not only structural, electrical, electromechanical, autonomic remodeling but also oxidative stress and inflammation [62–64].

A history of atrial fibrillation presupposes the existence of arrhythmogenic factors that are, as schematized by Philippe Coumel, electrophysiological substrate, trigger, and pejorative modulating factors [65]. In this field, which is already susceptible to arrhythmias, ACS can be formed as an arrhythmogenic substrate (the ischemic zone), as a modulating factor (by the discharge of catecholamines and by metabolic factors, such as ischemia and acidosis), and as a trigger factor (by accelerating the heart rate).

Arrhythmias, particularly atrial fibrillation, are well-known causes of stroke. The inverse is less well-known: indeed, damage to the central nervous system following a stroke often leads to a disturbance of the autonomic nervous system, which plays an important role in arrhythmogenesis [66]. Furthermore, neural necrosis activates a systemic inflammatory response, which also contributes to arrhythmogenesis [67].

Studies have found that dyslipidemia increases the risk of developing tachyarrhythmia in the acute phase of STEMI [68] and that patients with low HDL-C levels during hospitalization with ACS have an increased risk of developing cardiac rhythm disturbances, independent of other risk factors [69], suggesting a possible protective role of HDL-C against the onset of arrhythmias in the context of ACS. Indeed, HDL-C has been reported to have anti-inflammatory, antioxidant, and antithrombotic properties [70].

The rate of acute heart failure during STEMI reported in the literature ranges from 5% [53] to 20% [28] depending on the studies. The 13.1% frequency found in the current study falls within this range. Acute heart failure, as described by Killip and Kimball in 1967 [71], is one of the most frequent complications of STEMI. The occurrence of heart failure in the acute phase of ACS can be attributed to several intricate mechanisms, including microcirculatory dysfunction, myocardial hypoxia, myocytic necrosis, inflammation, hemorrhage, edema, decompensation of pre-existing heart failure, acute mitral regurgitation due to papillary muscle dysfunction, and remodeling [72]. It may also involve increased complement activation [73] and genetic factors [74].

The determinants of Killip ≥ 2 acute heart failure found in this study have also been found in other studies, as follows: older age [28], hypertension [75], and cigarette smoking [76].

Aging is a debilitating condition in which a conglomerate of cellular and molecular mechanisms underlies the effects of aging on cardiovascular function. The most important mechanisms are oxidative stress and low-grade chronic inflammation, superimposed on the limited capacity of cardiac regeneration; together, these processes promote the occurrence of heart failure in the dramatic context of ACS.

A recent study by Haig et al. has found that smoking independently predicted heart failure events after acute STEMI [76]. In a recent cohort of patients admitted for acute heart failure, most of whom had IHD, Dokoupil et al. have found that hypertension was the most common comorbidities [77]. Acar et al. have identified cigarette smoking as an independent predictor of deterioration in the LVEF in patients with STEMI [78]. Hypertension could increase the risk of acute heart failure probably by pre-existing hypertensive heart disease.

### Study limitations

Our study must be interpreted in the context of its potential limitations, including the limitations inherent in the cross-sectional study design, excluding the assessment of cause-and-effect relationships, and potential selection bias due to recruitment in a single hospital, thus excluding all cases that did not come to the CICU-SFHC or did not seek care during the study. Finally, generalizing the results to different populations is impossible.

## Conclusions

STEMI is a frequent condition at the SFHC and is often complicated by acute heart failure and arrhythmias. Patients aged ≥ 75 years, those with hypertension, those with diabetes mellitus, smokers, those with a history of atrial fibrillation or stroke, and those with low HDL-C levels require careful monitoring for early diagnosis and management of these complications.

## Data Availability

Because the consent given by study participants did not include data sharing with third parties, anonymized data can be made available to investigators for analysis on reasonable request to the corresponding author.

## Abbreviations

ACS: Acute coronary syndrome
AF: Atrial fibrillation
AIVR: Accelerated idioventricular rhythm
aOR: Adjusted odds ratio
AV bloc: Atrioventricular bloc
CI: Confidence interval
CICU: Cardiology Intensive Care Unit
CKD: Chronic kidney disease
COVID-19: Coronavirus disease 2019
CRP: C-reactive protein
CVDs: Cardiovascular diseases
CVRFs: Cardiovascular risk factors
D: Diagonal
DBP: Diastolic blood pressure
ESC: European Society of Cardiology
GFR: Glomerular filtration rate
HbA1c: Glycated haemoglobin
HbA1c: Glycated hemoglobin
HDL-C: High-density lipoprotein-cholesterol
IHD: Ischemic heart disease
K+: Potassium
LADA: Left anterior descending artery
LCA: Left coronary artery
LDL-C: Low-density lipoprotein cholesterol
LVEF: Left ventricular ejection fraction
MI: Myocardial infarction
MONICA: Monitoring of Trends and Determinants in Cardiovascular Disease
Na+: Sodium
non-STEMI: Non-ST-elevation myocardial infarction
OM: Obtuse marginal
ORs: Odds ratios
PAOD: Peripheral arterial occlusive disease
PDA: Posterior descending artery
PLA: Posterolateral artery
RCA: Right coronary artery
SBP: Systolic blood pressure
RI: Ramus intermedius
SFHC: Sud Francilien Hospital Center
SpO2: Oxygen saturation
STEMI: ST-elevation myocardial infarction
TIA: Transient ischemic attack
VF: Ventricular fibrillation
VSD: Ventricular septal defect
uVT: Unsustained ventricular tachycardia
